# Pro-/anti-inflammatory cytokine gene polymorphisms and chronic kidney disease: a cross-sectional study

**DOI:** 10.1186/1471-2369-13-2

**Published:** 2012-01-09

**Authors:** Rieko Okada, Kenji Wakai, Mariko Naito, Emi Morita, Sayo Kawai, Nobuyuki Hamajima, Megumi Hara, Naoyuki Takashima, Sadao Suzuki, Toshiro Takezaki, Keizo Ohnaka, Kokichi Arisawa, Hiroshi Hirohata, Keitaro Matsuo, Haruo Mikami, Michiaki Kubo, Hideo Tanaka

**Affiliations:** 1 Department of Preventive Medicine, Nagoya University Graduate School of Medicine, Nagoya, Japan; 2 Department of Preventive Medicine, Faculty of Medicine, Saga University, Saga, Japan; 3 Department of Health Science, Shiga University of Medical Science, Otsu, Japan; 4 Department of Public Health, Nagoya City University Graduate School of Medical Sciences, Nagoya, Japan; 5 Department of International Island and Community Medicine, Kagoshima University Graduate School of Medical and Dental Science, Kagoshima, Japan; 6 Department of Geriatric Medicine, Kyushu University Graduate School of Medical Sciences, Fukuoka, Japan; 7 Department of Preventive Medicine, Institute of Health Biosciences, University of Tokushima Graduate School, Tokushima, Japan; 8 Department of Epidemiology for Community Health and Medicine, Kyoto Prefectural University of Medicine, Kyoto, Japan; 9 Division of Epidemiology and Prevention, Aichi Cancer Center Research Institute, Nagoya, Japan; 10 Division of Cancer Registry, Prevention and Epidemiology, Chiba Cancer Center, Chiba, Japan; 11 Laboratory for Genotyping Development, Center for Genomic Medicine, RIKEN, Yokohama, Japan

## Abstract

**Background:**

The aim of this study was to explore the associations between common potential functional promoter polymorphisms in pro-/anti-inflammatory cytokine genes and kidney function/chronic kidney disease (CKD) prevalence in a large Japanese population.

**Methods:**

A total of 3,323 subjects aged 35-69 were genotyped for all 10 single nucleotide polymorphisms (SNPs) in the promoter regions of candidate genes with minor allele frequencies of > 0.100 in Japanese populations. The estimated glomerular filtration rate (eGFR) and CKD prevalence (eGFR < 60 ml/min/1.73 m^2^) of the subjects were compared among the genotypes.

**Results:**

A higher eGFR and lower prevalence of CKD were observed for the homozygous variants of *IL4 *-33CC (high IL-4 [anti-inflammatory cytokine]-producing genotype) and *IL6 *-572GG (low IL-6 [pro-inflammatory cytokine]-producing genotype). Subjects with *IL4 *CC + *IL6 *GG showed the highest mean eGFR (79.1 ml/min/1.73 m^2^) and lowest CKD prevalence (0.0%), while subjects carrying *IL4 *TT + *IL6 *CC showed the lowest mean eGFR (73.4 ml/min/1.73 m^2^) and highest CKD prevalence (17.9%).

**Conclusions:**

The functional promoter polymorphisms *IL4 *T-33C (rs2070874) and *IL6 *C-572G (rs1800796), which are the only SNPs that affect the IL-4 and IL-6 levels in Japanese subjects, were associated with kidney function and CKD prevalence in a large Japanese population.

## Background

Chronic kidney disease (CKD) is common and continues to increase. It is a risk factor for end-stage renal disease (ESRD) and is also a strong risk factor for cardiovascular diseases and mortality. A combined effect of environment and genotype determines the risk of CKD [1-3], and cytokine polymorphisms play important roles [3-5].

Cytokines are known to influence atherosclerosis, which causes CKD and subsequent ESRD [6,7]. The balance between pro- and anti-inflammatory cytokines determines the inflammatory response and may mediate the progression of CKD [6]. Among the cytokines, pro-inflammatory (IL-1, IL-6, and TNF-α) and anti-inflammatory (IL-4, IL-10, and IL-13) cytokines play pivotal roles [6]. IL-2 and IL-8 are also well-known pro-inflammatory cytokines that may affect CKD or ESRD progression [7,8]. Functional SNPs within the promoter area of these cytokine genes have been identified in that they influence the gene promoter activities and gene product levels [9,10]. Such polymorphisms have been demonstrated to be associated with susceptibility to a number of atherosclerotic diseases in CKD [3-5], but the issue of whether these cytokine polymorphisms are risk factors for CKD itself has not been fully clarified. Some studies have failed to show such associations, possibly owing to the small sample sizes, and their conclusions are controversial [11].

This study aimed to explore the associations between common potential functional promoter polymorphisms of pro-/anti-inflammatory cytokines and kidney function/CKD prevalence in a large Japanese population.

## Methods

### Study subjects

The study subjects were participants in the Japan Multi-Institutional Collaborative Cohort (J-MICC) Study, a large cohort study to confirm and detect gene-environment interactions in lifestyle-related diseases, in which voluntarily enrolled participants aged 35-69 from 10 areas of Japan provided blood and lifestyle data based on questionnaires. The details of the J-MICC Study have been described elsewhere [12]. The participants in this cross-sectional study were 4,519 subjects enrolled in the 10 study areas throughout Japan between 2004 and 2008 [13]. Serum creatinine (SCr) data were available in eight study areas. Of the 3,435 participants in these areas, 3,323 (97%) subjects were included in the analyses. Informed consent was obtained from all subjects and the study protocol was approved by the ethics committees of Nagoya University School of Medicine and the participating institutions.

### Selection and genotyping of polymorphisms

We selected pro-inflammatory (*IL1*, *IL2*, *IL6*, *IL8*, and *TNFA*) and anti-inflammatory (*IL4*, *IL10*, and *IL13*) cytokine genes as candidate genes based on the published literature [6-8]. In addition, *CD14*, which encodes a lipopolysaccharide receptor that initiates the inflammatory response, was selected based on our previous report [14]. Subsequently, to the best of our knowledge, we selected all the single nucleotide polymorphisms (SNPs) in the promoter regions that had minor allele frequencies in Japanese populations of > 0.100, based on the database SNP (dbSNP) and the HapMap database, and that had potential functional effects according to the published literature. The selected SNPs were *IL4 *T-33C (rs2070874, which is in complete linkage disequilibrium with T-589C [rs2243250]) [9,15], *IL6 *C-572(-634)G (rs1800796) [10,11], *IL1B *T-31C (rs1143627, linked to C-511T [rs16944]), *IL2 *T-330G (rs2069762), *IL13 *C-1111T (rs1800925) [16], and *CD14 *A-260(-159)G (rs2569190) [14], which were proven to be functional, and *IL8 *T-251A (rs4073), *IL10 *T-819C (rs1800871, linked to A-592C [rs1800872]), *TNFA *C-857T (rs1799724), and *TNFA *T-1031C (rs1799964, linked to C-863A [rs1800630]), which were reported to be probable or presumed functional SNPs [16,17]. *IL6 *T-6331C (rs10499563), A-597G (rs1800797), C-174G (rs1800795), and *IL10 *A-1082G (rs1800896) were excluded because their minor allele frequencies were < 0.100.

DNA was extracted from buffy coat fractions using a BioRobot M48 Workstation (Qiagen Group, Tokyo, Japan), or from whole blood using an automatic nucleic acid isolation system (NA-3000; KURABO, Osaka, Japan). The SNPs were genotyped using a Multiplex PCR-based Invader Assay (Third Wave Technologies, Madison, WI) [18] at the Laboratory for Genotyping Development, Center for Genomic Medicine, RIKEN. The genotype call rates were 99.40% to 99.98%.

### Estimated glomerular filtration rate (eGFR), and definitions of CKD and comorbid diseases

SCr was measured in all participants using an enzymatic method. The eGFR of each participant was calculated from the SCr, age, and sex using the following Japanese eGFR equation recently determined by the Japanese Society of Nephrology [19]:

$$\text{eGFR }\left(\text{ml}∕\text{min}∕\text{1}.\text{73}{\text{m}}^{\text{2}}\right)=\text{ 194}\times \text{ SCr }{\left(\text{mg}∕\text{dl}\right)}^{-\text{1}.0\text{94}}\times \text{ ag}{\text{e}}^{-0.\text{287}}\left(\times 0.\text{739 if female}\right).$$

The prevalence of CKD was determined for CKD stages 3-5 (defined as eGFR < 60 ml/min/1.73 m^2^) [20].

Hypertension was defined as resting blood pressure ≥ 140/90 mmHg or being under treatment for hypertension. Diabetes mellitus was defined as fasting blood glucose ≥ 126 mg/dl or serum HbA1c ≥ 6.5% or being under treatment for diabetes mellitus.

### Statistical analysis

The genotype distributions were tested for Hardy-Weinberg equilibrium. One-way analysis of variance (ANOVA) or the χ^2 ^test was used for comparisons of mean eGFRs and CKD prevalences between genotypes, and the polymorphisms with a significant difference in the mean eGFR or CKD prevalence were selected for multivariate analyses. The mean eGFRs, adjusted for age (continuous variable), sex, comorbid hypertension and diabetes mellitus, and history of cardiovascular diseases by multiple linear regression models, were compared between genotypes using the homozygous for the major allele as the reference. Odds ratios (ORs) for CKD prevalence adjusted for these covariates were estimated by unconditional logistic regression analysis with 95% confidence intervals (CIs). Standard errors were adjusted for the study areas. The analyses were carried out using STATA ver. 9 software (StataCorp, College Station, TX).

## Results

The characteristics and the genotype frequencies of the subjects are summarised in Table 1 and Table 2. Seven subjects had a history of kidney disease, and two were categorised into the CKD group. The genotype frequencies were in Hardy-Weinberg equilibrium (data not shown), except for *IL6 *C-572G (57.5% for CC, 35.7% for CG, and 6.8% for GG, P = 0.026, in comparison with the expected values of 57.2%, 36.9%, and 5.9%, respectively) [13].

**Table 1:** Clinical characteristics of study subjects

	CKD	Non-CKD	Total
	(n = 546)	(n = 2,777)	(n = 3,323)
Age (years)	60.6 ± 7.2	55.9 ± 8.7	56.7 ± 8.6
Male	252(46.2%)	1,364(49.1%)	1,616(48.6%)
Body mass index	23.5 ± 3.1	23.4 ± 3.3	23.4 ± 3.3
Hypertension	245(44.9%)	1,050(37.8%)	1,295(39.0%)
Systolic blood pressure (mm Hg)	130.5 ± 19.8	128.1 ± 19.3	128.5 ± 19.4
Diastolic blood pressure (mm Hg)	79.1 ± 12.4	78.6 ± 11.9	78.7 ± 12.0
Anti-hypertensive medication	146(26.7%)	503(18.1%)	649(19.5%)
Diabetes mellitus	54(9.9%)	220(7.9%)	274(8.2%)
Fasting plasma glucose (mmol/l)	5.49 ± 1.23	5.55 ± 1.17	5.54 ± 1.17
HbA1c (%)	5.22 ± 0.69	5.22 ± 0.66	5.22 ± 0.67
Glucose-lowering medication	28(5.1%)	117(4.2%)	145(4.4%)
Cardiovascular diseases	34(6.2%)	80(2.9%)	114(3.4%)
Total cholesterol (mmol/l)	5.66 ± 0.88	5.46 ± 0.83	5.50 ± 0.88
HDL cholesterol (mmol/l)	1.60 ± 0.41	1.64 ± 0.42	1.63 ± 0.42
Lipid-lowering medication	68(12.5%)	233(8.4%)	301(9.1%)
Uric acid (μmol/l)	333 ± 89	303 ± 77	309 ± 83
Current smokers	68(12.5%)	495(17.8%)	563(16.9%)

Results are expressed as mean ± SD or number (%). CKD = chronic kidney disease. CKD is defined by eGFR < 60 ml/min/1.73 m^2^. Hypertension = blood pressure ≥ 140/90 mmHg or under anti-hypertensive medication. Diabetes mellitus = fasting blood glucose ≥ 126 mg/dl, HbA1c ≥ 6.5% or under glucose-lowering medication.

**Table 2:** Genotype frequencies of study subjects

Genotype	CKD	non-CKD	Total
	(n = 546)	(n = 2,777)	(n = 3,323)
*IL1B *T-31C (rs1143627)			
T T	163 (29.9%)	791 (28.5%)	954 (28.7%)
C T	266 (48.7%)	1,355 (48.8%)	1,621 (48.8%)
C C	117 (21.4%)	631 (22.7%)	748 (22.5%)
*IL2 *T-330G (rs2069762)			
T T	244 (44.9%)	1,225 (44.1%)	1,469 (44.2%)
T G	241 (44.3%)	1,239 (44.6%)	1,480 (44.6%)
G G	59 (10.9%)	312 (11.2%)	371 (11.2%)
*IL4 *T-33C (rs2070874)			
T T	259 (47.4%)	1,193 (43.0%)	1,452 (43.7%)
T C	241 (44.1%)	1,226 (44.2%)	1,467 (44.2%)
C C	46 (8.4%)	357 (12.9%)	403 (12.1%)
*IL6 *C-572G (rs1800796)			
C C	312 (57.1%)	1,600 (57.6%)	1,912 (57.6%)
G C	208 (38.1%)	977 (35.2%)	1,185 (35.7%)
G G	26 (4.8%)	199 (7.2%)	225 (6.8%)
*IL8 *T-251A (rs4073)			
T T	254 (46.5%)	1,281 (46.5%)	1,535 (46.5%)
A T	235 (43.0%)	1,204 (43.7%)	1,439 (43.6%)
A A	57 (10.4%)	273 (9.9%)	330 (10.0%)
*IL10 *T-819C (rs1800871)			
T T	252 (46.2%)	1,175 (42.5%)	1,427 (43.1%)
C T	239 (43.8%)	1,222 (44.2%)	1,461 (44.1%)
C C	55 (10.1%)	370 (13.4%)	425 (12.8%)
*IL13 *C-1111T (rs1800925)			
C C	370 (67.9%)	1,847 (66.5%)	2,217 (66.8%)
T C	158 (29.0%)	840 (30.3%)	998 (30.1%)
T T	17 (3.1%)	89 (3.2%)	106 (3.2%)
*TNFA *C-857T (rs1799724)			
C C	373 (68.3%)	1,788 (64.4%)	2,161 (65.0%)
C T	155 (28.4%)	887 (31.9%)	1,042 (31.4%)
T T	18 (3.3%)	102 (3.7%)	120 (3.6%)
*TNFA *T-1031C (rs1799964)			
T T	383 (70.2%)	1,934 (69.6%)	2,317 (69.7%)
C T	144 (26.4%)	764 (27.5%)	908 (27.3%)
C C	19 (3.5%)	79 (2.8%)	98 (2.9%)
*CD14 *T-260C (rs2569190)			
T T	144 (26.4%)	785 (28.3%)	929 (28.0%)
T C	275 (50.4%)	1,412 (50.9%)	1,687 (50.8%)
C C	127 (23.3%)	580 (20.9%)	707 (21.3%)

Results are expressed as number (%). CKD = chronic kidney disease. CKD is defined by eGFR < 60 ml/min/1.73 m^2^.

The mean eGFRs and CKD prevalences were compared among the genotypes of the 10 cytokine SNPs (Table 3). The mean eGFRs differed for the *IL4 *T-33C (rs2070874) and *IL6 *C-572G (rs1800796) genotypes (P = 0.012 and P = 0.004, respectively), while the CKD prevalences differed for the *IL4 *T-33C genotypes. Thus, the *IL4 *and *IL6 *genotypes were subjected to multivariate analyses.

**Table 3:** Mean eGFRs and CKD prevalence with respect to cytokine polymorphism genotypes

Genotype	n	eGFR (ml/min/1.73 m^2^)			CKD (eGFR < 60 ml/min/1.73 m^2^)		
				
		mean ± SD	P-value^†^		n	(%)	P-value^‡^
*IL1B *T-31C (rs1143627)							
T T	954	74.0 ± 14.9			163	(16.6%)	
C T	1,621	73.9 ± 14.6	0.576		266	(16.3%)	0.727
C C	748	74.6 ± 15.3			117	(15.6%)	
*IL2 *T-330G (rs2069762)							
T T	1,469	74.0 ± 14.8			244	(16.6%)	
T G	1,480	74.2 ± 14.9	0.860		241	(16.3%)	0.938
G G	371	74.3 ± 14.5			59	(15.9%)	
*IL4 *T-33C (rs2070874)							
T T	1,452	73.4 ± 14.6			259	(17.8%)	
T C	1,467	74.4 ± 15.1	**0.012**		241	(16.4%)	**0.009**
C C	403	75.8 ± 14.5			46	(11.4%)	
*IL6 *C-572G (rs1800796)							
C C	1,912	74.2 ± 14.7			312	(16.3%)	
G C	1,185	73.4 ± 14.8	**0.004**		208	(17.6%)	0.082
G G	225	76.9 ± 15.9			26	(11.6%)	
*IL8 *T-251A (rs4073)							
T T	1,535	74.1 ± 14.4			254	(17.3%)	
A T	1,439	73.9 ± 15.2	0.745		235	(16.3%)	0.917
A A	330	74.6 ± 15.4			57	(16.5%)	
*IL10 *T-819C (rs1800871)							
T T	1,427	73.6 ± 14.7			252	(17.7%)	
C T	1,461	74.2 ± 14.8	0.155		239	(16.4%)	0.070
C C	425	75.2 ± 15.0			55	(12.9%)	
*IL13 *C-1111T (rs1800925)							
C C	2,217	74.0 ± 14.9			370	(16.7%)	
T C	998	74.3 ± 14.7	0.734		158	(15.8%)	0.827
T T	106	74.7 ± 14.3			17	(16.0%)	
*TNFA *C-857T (rs1799724)							
C C	2,161	73.6 ± 14.7			373	(17.3%)	
C T	1,042	74.9 ± 15.0	0.054		155	(14.9%)	0.212
T T	120	75.5 ± 15.4			18	(15.0%)	
*TNFA *T-1031C (rs1799964)							
T T	2,317	73.9 ± 14.6			383	(16.5%)	
C T	908	74.5 ± 15.4	0.641		144	(15.9%)	0.652
C C	98	74.1 ± 14.6			19	(19.4%)	
*CD14 *T-260C (rs2569190)							
T T	929	74.5 ± 14.5			144	(15.5%)	
T C	1,687	74.2 ± 14.9	0.226		275	(16.3%)	0.403
C C	707	73.3 ± 14.9			127	(18.0%)	

^†^P for ANOVA, ^‡^P for the χ^2 ^test. eGFR = estimated glomerular filtration rate. CKD = chronic kidney disease. CKD is defined by eGFR < 60 ml/min/1.73 m^2^. Bold style represents P < 0.05.

Higher eGFRs and lower CKD prevalences were observed for the *IL4 *-33CC and *IL6 *-572GG genotypes (Table 4). The mean eGFRs were 75.7 and 73.4 ml/min/1.73 m^2 ^for the *IL4 *CC and TT genotype carriers, and 76.9 and 74.2 ml/min/1.73 m^2 ^for the *IL6 *GG and CC genotype carriers, respectively. The CKD prevalences were 11.4% and 17.8% for the *IL4 *CC and TT genotype carriers (OR = 0.59, 95% CI = 0.37-0.95, P = 0.029 after adjustment), and 11.6% and 16.3% for the *IL6 *GG and CC genotype carriers (OR = 0.67, 95% CI = 0.50-0.90, P = 0.008 after adjustment), respectively. These differences were greater when the two genotypes were combined (Table 5). Subjects with both the *IL4 *CC and *IL6 *GG genotypes showed the highest mean eGFR (79.1 ml/min/1.73 m^2^) and lowest CKD prevalence (0.0%), while subjects carrying both the *IL4 *TT and *IL6 *CC genotypes showed the lowest mean eGFR (73.4 ml/min/1.73 m^2^) and highest CKD prevalence (17.9%). There was no interaction between the *IL4 *CC and *IL6 *GG genotypes, and their effects were additive.

**Table 4:** Mean eGFRs and CKD prevalence for ***IL4 ***T-33C and ***IL6 ***C-572G genotypes

Genotype		n	eGFR (ml/min/1.73 m^2^)					CKD (eGFR < 60 ml/min/1.73 m^2^)				
					
			mean ± SD	β^†^	(95% CI)	P-value^†^		n	(%)	OR^†^	(95% CI)	P-value^†^
*IL4 *T-33C												
	TT	1,452	73.4 ± 14.6	0	(reference)	-		259	(17.8%)	1	(reference)	-
	TC	1,466	74.4 ± 15.1	0.9	(-1.2-2.9)	0.346		241	(16.4%)	0.91	(0.78-1.07)	0.269
	**CC**	403	75.7 ± 14.5	2.2	(-1.9-6.3)	0.240		46	(11.4%)	**0.59**	(0.37-0.95)	0.029
*IL6 *C-572G												
	CC	1,911	74.2 ± 14.7	0	(reference)	-		312	(16.3%)	1	(reference)	-
	CG	1,185	73.4 ± 14.8	-1.1	(-2.3-0.1)	0.065		208	(17.6%)	1.13	(0.98-1.31)	0.091
	**GG**	225	76.9 ± 15.9	2.6	(-0.1-5.2)	0.055		26	(11.6%)	**0.67**	(0.50-0.90)	0.008

^†^adjusted for age, sex, hypertension, diabetes mellitus, and cardiovascular diseases. Standard errors were adjusted for study areas. eGFR = estimated glomerular filtration rate. CKD = chronic kidney disease. CKD is defined by eGFR < 60 ml/min/1.73 m^2^.

**Table 5:** Mean eGFRs and CKD prevalence for ***IL4 ***T-33C and ***IL6 ***C-572G genotypes combined

Genotype		n	eGFR (ml/min/1.73 m^2^)					CKD (eGFR < 60 ml/min/1.73 m^2^)				
					
			mean ± SD	β^†^	(95% CI)	P-value^†^		n	(%)	OR^†^	(95% CI)	P-value^†^
*IL4 *TT/	*IL6 *CC	849	73.4 ± 14.4	0	(reference)	-		152	(17.9%)	1	(reference)	-
*IL4 *TC/	*IL6 *CC	854	74.5 ± 14.9	1.1	(-0.3-2.4)	0.108		140	(16.4%)	0.89	(0.75-1.06)	0.200
*IL4 *TT/	*IL6 *CG	512	72.5 ± 14.5	-0.8	(-2.8-1.1)	0.337		98	(19.1%)	1.08	(0.90-1.30)	0.387
*IL4 *TC/	*IL6 *CG	512	74.0 ± 15.2	0.2	(-3.1-3.5)	0.890		84	(16.4%)	0.94	(0.68-1.28)	0.686
*IL4 ***CC **/	*IL6 *CG	161	74.0 ± 14.4	0.3	(-3.8-4.4)	0.867		26	(16.1%)	0.89	(0.42-1.90)	0.768
*IL4 *TC/	*IL6 ***GG**	100	75.6 ± 17.0	2.1	(-1.5-5.7)	0.217		17	(17.0%)	0.94	(0.67-1.32)	0.720
*IL4 ***CC **/	*IL6 *CC	208	76.5 ± 14.6	3.1	(-1.2-7.4)	0.127		20	(9.6%)	**0.48**	(0.39-0.58)	< 0.001
*IL4 *TT/	*IL6 ***GG**	91	77.6 ± 15.4	**4.2**	(0.4-8.1)	0.036		9	(9.8%)	**0.49**	(0.37-0.63)	< 0.001
*IL4 ***CC **/	*IL6 ***GG**	34	79.1 ± 13.4	**5.2**	(0.3-10.2)	0.041		0	(0.0%)	**0.00**	-	-

^†^adjusted for age, sex, hypertension, diabetes mellitus, and cardiovascular diseases. Standard errors were adjusted for study areas. eGFR = estimated glomerular filtration rate. CKD = chronic kidney disease. CKD is defined by eGFR < 60 ml/min/1.73 m^2^.

We previously reported an association between the *CD14 *A-260G SNP and kidney function among a population living in the north part of Japan using an eGFR derived from the MDRD Study equation [14]. However, the present study did not show such an association.

## Discussion

Our explorations revealed that the subjects with the genotypes *IL4 *-33CC (a genotype that produces high levels of IL-4) and *IL6 *-572GG (a genotype that produces low levels of IL-6) had better kidney function and a lower risk of CKD in a large Japanese population.

Cytokines are important modulators of inflammation, and the balance between pro- and anti-inflammatory cytokines determines the inflammatory response and may mediate the progression of atherosclerosis and subsequent CKD [6,7]. Genetic polymorphisms of these cytokines have been shown to be associated with comorbidities, such as cardiovascular disease, in ESRD patients [3-5], or with ESRD susceptibility [8], but there are controversial results that no polymorphisms of the *IL6*, *IL10*, and *IL1 *genes were associated with ESRD [11]. The evidence for polymorphisms in cytokine genes affecting the risk of CKD itself is scarce and this issue has not been fully clarified, especially in the general population.

IL-4 is an anti-inflammatory cytokine that exerts immunosuppressive effects on macrophages and suppresses pro-inflammatory cytokine production [21]. The promoter polymorphism *IL4 *T-33C (which is in complete linkage disequilibrium with *IL4 *T-589C) affects IL-4 expression [15], and the CC genotype shows a high level of IL-4 protein [9]. Accordingly, subjects with the CC genotype showed a lower risk of ischemic stroke relapse [22], consistent with our data indicating a lower risk of CKD in CC genotype carriers.

An elevated level of the main pro-inflammatory cytokine IL-6 predicts cardiovascular mortality in ESRD patients [3]. The *IL6 *C to G variation at position -572 reduces the transcriptional activity of the *IL6 *promoter, and the levels of IL-6 are lower in carriers of the *IL6 *-572GG genotype [10,11]. Accordingly, the *IL6 *-572GG genotype was associated with lower risks of kidney allograft survival [23] and abdominal aortic aneurysm [24], consistent with our data showing a lower risk of CKD in GG genotype carriers.

The combined effect of high IL-4- and low IL-6-producing genotypes has been shown to lead to a lower risk of ESRD [8]. We also found that no CKD subjects carried high IL-4- and low IL-6-producing (low-risk) genotypes, and that their mean eGFR was 5.2 ml/min/1.73 m^2 ^higher than that in carriers of the low IL-4- and high IL-6-producing (high-risk) genotypes. This difference is almost equivalent to a 14-year difference in a healthy Japanese population [25], and has a significant impact with respect to cardiovascular disease prevention, especially among healthy individuals who are not aware of a possible risk of CKD.

The evidence for polymorphisms in cytokine genes affecting the risk of CKD is scarce. No cytokine genes were identified as susceptibility loci for CKD in a Caucasian population in a genome-wide association study (GWAS) [1]. However, this may simply mean that no cytokine genes were highly statistically significantly associated in the context of the multiple testing related to the GWAS, and therefore not presented in the GWAS report. Yoshida *et al*. [2] showed that some genetic variants were associated with CKD in a large Japanese population, but only *TNFA *and *IL10 *were included as representative cytokine genes and did not show associations. In contrast, we selected candidate genes that are assumed to be of physiological interest based on the associations between pro-/anti-inflammatory cytokines and CKD [6,7]. The other difference is that the former study participants were mixed, and comprised patients with various symptoms, health check-up examinees and aged subjects [2]. In contrast, our study participants were enrolled from the population with an age range of 35 to 69. Thus, our study contains the largest general Japanese population investigated to date for associations among pro-/anti-inflammatory cytokine gene variants and CKD.

No haplotype analyses were needed because *IL4 *T-33C and *IL6 *C-572G are the only SNPs that affect the IL-4 and IL-6 levels in Japanese subjects. *IL4 *T-589C, *IL4 *T-33C, and a 70-bp variable number of tandem repeat polymorphism (VNTR) within intron 3 are in complete linkage disequilibrium, thus there are only two *IL4 *gene haplotypes in Japanese populations; -589T/-33T/B1 (183 bp) (allele frequency, 0.670) and -589C/-33C/B2 (253 bp) (0.330) [15]. In addition, *IL6 *transcription is influenced by four promoter polymorphisms (C-572G, A-597G, -373AnTn and C-174G) [26], but A-597G and C-174G do not exist or are very rare in Japanese populations. Only three prevalent haplotypes have been identified in Japanese populations [10]: -572C/-373A10T10 (allele frequency 0.733), G/A10T11 (0.136), and G/A9T11 (0.104). The serum levels of IL-6 were high in C/A10T10 and low in both G/A10T11 and G/A9T11, with no difference between the IL-6 levels in G/A10T11 and G/A9T11. Thus, *IL4 *T-33C and *IL6 *C-572G could be the only SNPs that affect the transcriptional activity of IL-4 and IL-6 in Japanese populations.

A limitation of our study was that *IL6 *C-572G was not in Hardy-Weinberg equilibrium. However, the absolute difference between the actual and expected frequencies was only 1%. Thus, the errors in genotyping seem unlikely to result in substantial misclassification [13], although the large number in the study population might account for the statistically significant deviation from Hardy-Weinberg equilibrium. Another limitation is the possible misclassification of CKD subjects owing to the single measurement of SCr. As our subjects were presumably healthy volunteers or health check-up examinees, their kidney function might have been stable. Thus, the possibility that a single measurement of SCr could result in misclassification is low. Since we lacked a replication cohort, adjustment of the p-values for the multiple comparisons, and biomarkers of inflammation, further confirmation using another cohort of subjects is needed so that there is a high chance that our study results represent a false positive finding. As this was a cross-sectional study, a prospective study is needed to further confirm the decrease in eGFR and the incident risk of CKD.

## Conclusions

The functional cytokine polymorphisms *IL4 *T-33C (rs2070874) and *IL6 *C-572G (rs1800796), which are the only SNPs identified to date that affect the IL-4 and IL-6 levels in Japanese subjects, were associated with kidney function and CKD prevalence in a large Japanese population. The *IL4 *-33CC and *IL6 *-572GG genotypes (high IL-4-and low IL-6-producing genotypes) showed better kidney function and a lower risk of CKD compared with the other variants. These differences were greater when the two genotypes were combined. This study supported the hypothesis that genetic variations in the anti-inflammatory cytokine *IL4 *and pro-inflammatory cytokine *IL6 *genes may predispose subjects to the development of CKD. Determination of the genotypes involved may prove informative for identifying individuals at a lower or higher risk of CKD. Further confirmation in another study is needed.

## Competing interests

The authors declare that they have no competing interests.

## Authors' contributions

RO analyzed data and mainly drafted the article. KW, MN, KA, and MK were involved in drafting the manuscript and revising it critically for important intellectual content. EM, SK, MH, NT, SS, TT, KO, HH, KM, and HM made substantial contributions to the conception, design, and acquisition of data. NH and HT initiated the study and gave final approval of the version to be published. All the authors have read and approved the final manuscript.

## Pre-publication history

The pre-publication history for this paper can be accessed here:

http://www.biomedcentral.com/1471-2369/13/2/prepub
